# Editors’ page

**DOI:** 10.1016/j.ajpc.2021.100297

**Published:** 2021-11-22

**Authors:** Erin D. Michos, Nathan D. Wong

**Affiliations:** a Division of Cardiology, Johns Hopkins University School of Medicine, Baltimore, MD USA; b Division of Cardiology, University of California Irvine School of Medicine, C240 Medical Sciences, University of California, Irvine, CA 92697, USA

We are proud to announce with this issue that we have now completed two years of publication of the *American Journal of Preventive Cardiology.* We cannot express how grateful we are to all of you who have given the *Journal* the opportunity to publish your work, as well as our dedicated editorial board and associate editors, the leadership of the American Society for Preventive Cardiology (ASPC), and our publisher Elsevier. You all believed in the promise and potential of the *Journal* from the beginning, and without your support and effort, the *Journal*’s productivity to this point would have not been possible. The acceptance and indexing of the *Journal* into Pub Med Central earlier this year, barely a year after its inception, is testament to its importance in publishing the best work in preventive cardiology. The *Journal* has received to date well over 200 submissions, including more than 125 original contributions and more than 30 state-of-the-art reviews, with an average time to first decision of 2.5 weeks.

This 8th issue of the *Journal* again brings more content to advance the prevention field forward. The issue begins with unique ASPC practice statement on enrollment of women and diverse populations in cardiovascular clinical trials and a commentary on disparities in COVID-19 and implications for preventive cardiology. Some original research articles of note include one on age and sex disparities in hypertension control in the Multiethnic Study of Atherosclerosis, how thoracic aortic calcification may improve risk prediction and help inform statin eligibility, a simulation analysis of cost effectiveness of genetic testing in familial hypercholesterolemia, as well as one on the interplay of sleep duration and inflammation on cardiovascular mortality. Other original contributions also nicely round out the issue as well as a state-of-the-art review on a paradox of atrial fibrillation involving hypertension and stroke.

As we close in on the Holidays, we again thank all of you who have contributed to the success of the *Journal*, and wish you all a safe and successful New Year. With the COVID-19 pandemic hopefully coming in better control, we hope to see many of you in the near future!

